# Association of term isolated microcephaly with mode of delivery and perinatal outcome - a retrospective case-control analysis

**DOI:** 10.1186/s12884-021-03613-y

**Published:** 2021-02-09

**Authors:** Ron Bardin, Eyal Krispin, Lina Salman, Inbal Navon, Anat Shmueli, Sharon Perlman, Yinon Gilboa, Eran Hadar

**Affiliations:** 1 Helen Schneider Hospital for Women, Rabin Medical Center, Petach Tikva; affiliated to Sackler Faculty of Medicine, Tel Aviv University, Tel Aviv, Israel; 2 Hillel Yaffe Medical Center, Hadera; affiliated to Rappaport Faculty of Medicine, Technion, Haifa, Israel

**Keywords:** Ultrasound, Fetal microcephaly, Mode of delivery, Fetal outcome

## Abstract

**Background:**

We aimed to evaluate the association of isolated fetal microcephaly measured by ultrasound prior to delivery at term with mode of delivery and perinatal outcome.

**Methods:**

A single-center retrospective study was conducted in 2012–2016. Fetal microcephaly was defined as head circumference > 2 standard deviations of the mean for gestational age and sex. We compared the obstetric, delivery, and outcome parameters of women in whom ultrasound performed up to 10 days prior to term delivery showed isolated fetal microcephaly (study group) or normal head circumference (reference group). Exclusion criteria were intrauterine fetal death, birthweight below the 10th percentile, and antepartum cesarean delivery for any indication.

**Results:**

Of 3677 women included in the study, 26 (0.7%) had a late ultrasound finding of isolated fetal microcephaly. Baseline characteristics were similar in the two groups except for estimated fetal weight based on abdominal circumference and biparietal diameter, which was lower in the microcephaly group (3209.8 ± 557.6 vs. 2685.8 ± 420.8 g, *p* < .001). There was no significant between-group difference in rate of vaginal operative deliveries (11.7% vs 14.8%, respectively, *p* = 0.372). The study group had no intrapartum cesarean deliveries compared to 6.3% of the reference group (NS). Compared to controls, neonates in the study group were smaller (3323.2 ± 432.2 vs. 2957.0 ± 330.4 g, *p* < .001), with lower birthweight percentile (60.5 ± 26.5 vs. 33.6 ± 21.5%, *p* < .001) and were more often males (48.2 vs. 90.0%, *p* < .001). No significant differences were noted in perinatal outcomes between the groups, including admission to neonatal intensive care unit, intraventricular hemorrhage, 5-min Apgar score < 7, asphyxia, seizures, and sepsis.

**Conclusions:**

Isolated microcephaly in term fetuses is not advantageous for a vaginal delivery, nor does it does not pose a greater than normal risk of adverse perinatal outcome.

## Background

Microcephaly, or small head circumference, is a neurologic marker which can be detected antenatally during routine sonographic examination or post-partum, during neonatal physical examination. Its prevalence is relatively low, reported at 0.1 to 0.5% of children. It is a challenging issue owing to its difficult diagnosis, various cutoffs for definition, and complexity to ascertain its etiology. It has heterogenous prognosis depending on its severity, etiology and associated findings [1].

Postnatally, microcephaly is usually defined as an occipitofrontal circumference of at least 2 standard deviations (SD) below the mean for age and sex, but some authors use a cutoff of below 3SD [1–3]. Head circumference (HC) below 3SD of the mean is more likely to be associated with disorders, either genetic or nongenetic, affecting brain development and consequent intellectual disability and neurological abnormalities [4–6]. However, it is commonly agreed that prenatally, targeted evaluation for fetal microcephaly should be initiated when HC is at or below 2SD of the mean [7, 8]. There is often a discrepancy between prenatal and postnatal measurements of HC owing to changes in head diameter when the fetus passes through the bony pelvis during labor (e.g., molding) [4, 9] and the different measuring techniques used in the two settings [4, 8].

The basic premise underlying the concept of cephalopelvic disproportion is that protracted or arrested labor results from a mismatch between the maternal pelvis and the fetal head proportions [10]. Fetal head dimensions have an impact on the risk of operative delivery and maternal and fetal complications. Increased bi-parietal diameter (BPD) measured up to 7 days before labor in term fetuses was found to be associated with a significantly higher rate of operative vaginal delivery (OVD), without adverse neonatal outcomes. Large HC was found to be an independent risk factor for OVD as well as unplanned cesarean delivery (CD) [9, 11–13].

However, the current literature on fetal microcephaly mostly focuses on the prenatal diagnostic work-up to detect its etiology. The prognosis depends on the type of microcephaly (isolated or syndromic), whether microcephaly is primary or secondary, and the causative factor(s) - genetic, infectious, or environmental [4–8, 14, 15]. Data are lacking on the association between microcephaly and labor and delivery outcome, and it remains unclear if microcephaly facilitates vaginal delivery or poses a risk of adverse outcome during passage of the fetus in the birth canal.

We hypothesized that microcephaly will be associated with higher rates of vaginal delivery, but the small head circumference may be more vulnerable to adverse outcome. Therefore, the aim of the present study was to evaluate the potential association of isolated fetal microcephaly measured by ultrasound prior to delivery with mode of delivery and perinatal outcomes.

## Methods

### Study population

The healthcare database of a tertiary university-affiliated medical center was searched for all women who gave birth from July 2012 to December 2016. Inclusion criteria were term birth (at or beyond 37 + 0 gestational weeks), singleton liveborn infant, absence of fetal genetic abnormalities or major structural malformations, and available sonographic measurement of fetal head circumference up to 10 days prior to delivery. Exclusion criteria were intrauterine fetal death, fetal head circumference above the 95th percentile for gestational age or birthweight below the 10th percentile, and antepartum cesarean delivery for any indication.

Eligible women were divided into two groups on the basis of antepartum fetal head circumference: Group 1 (cases) - at or below 2SD of the mean for gestational age and sex (isolated microcephaly group); 2) Group 2 (control) - normal head circumference for gestational age and sex (reference group).

### Data collection

Data were retrieved from the hospital’s comprehensive computerized maternal, fetal, and neonatal medical records and included demographics, medical and obstetrical background, antepartum sonographic biometric measurements, birth and perinatal outcomes.

All sonograms were performed in our dedicated obstetrical ultrasound unit by expert sonographers or experienced ultrasound technicians. Measurements made by ultrasound technicians were reviewed and confirmed by a senior physician. Sonographic examinations were performed using the Voluson E8 (GE Medical systems, Kretz Ultrasound, Zipf, Austria) equipped with a Trans-vaginal transvaginal transducer of 5 to 9 MHz and Trans-Abdominal curvilinear Transducer of 4–8 MHz.

### Definitions

Gestational age was estimated on the basis of the last menstrual period and affirmed by first trimester crown-rump length ultrasound measurement.

Microcephaly was defined as head circumference at or below 2SD of the mean for age and sex according to the reference charts of Chervenak et al. [4].

Birth weight percentile was calculated using gender-specific, population-based birthweight curves [16].

Small for gestational age was defined as birthweight below the 10th percentile.

Grade 1 or 2 perineal tear or episiotomy was considered a minor laceration, and grade 3 or 4 perineal tear was categorized as obstetric anal sphincter injury syndrome (OASIS).

Neonatal diagnoses were assigned by the treating pediatrician in the neonatal nursery unit or the neonatal intensive care unit (NICU) according to international and local guidelines.

### Outcome measures

The primary outcome measure of the study was mode of delivery: vaginal (spontaneous or operative) or intrapartum cesarean, stratified by indication (non-reassuring fetal heart rate, arrested/protracted labor, or failed vaginal operative delivery). The secondary outcome measures were maternal adverse events (episiotomy and OASIS), neonatal adverse events (NICU admission, intraventricular hemorrhage, and respiratory distress syndrome or transient tachypnea of the newborn), as well as composite outcome (i.e., presence of any one of the following: NICU admission, intraventricular hemorrhage, 5-min Apgar score < 7, asphyxia, seizures, and sepsis).

### Statistical analysis

Statistical analysis was performed using SAS software, version 9.4 (SAS Institute, Cary, North Carolina USA). Descriptive statistics are presented by number and percentage for categorical variables and by mean and standard deviation for continuous variables. Categorical variables were compared by chi-square or Fisher exact test, as appropriate, and continuous variables, by t-test. A *p-*value < 0.05 was considered significant.

## Results

Of 5115 women who met the inclusion criteria, 1438 were excluded because of antepartum cesarean delivery (*n* = 1101), small for gestational age infant (*n* = 331) and intrauterine fetal death (*n* = 6). The remaining 3677 women formed the study group.

The fetal ultrasound performed up to 10 days before delivery showed a normal head circumference in 3651 cases (99.3%) and microcephaly in 26 (0.7%). The demographic, medical, and obstetric characteristics of the two groups are presented in Table 1. As expected, the microcephaly group had a significantly smaller fetal head circumference than the reference group (306.1 ± 10.1 vs 329.2 ± 15.4 mm, *p* < .001) and smaller biparietal diameter (85.4 ± 3.8 vs 91.3 ± 5.0 mm, *p* < .001) in addition to a lower estimated fetal weight based on abdominal circumference and biparietal diameter (2685.8 ± 420.8 vs 3209.8 ± 557.6 g, *p* < .001). There were no other significant differences between the groups.

**Table 1:** Demographic, medical, and obstetrical parameters in 3677 fetuses stratified by head circumference

Parameter	Normal HC	Microcephaly	***p***-Value
N	**3651**	**26**	
Age, years	31.3 ± 5.0	32.3 ± 4.5	0.472
Gravidity	2.7 ± 1.8	3.2 ± 2.9	0.359
Parity	1.2 ± 1.4	1.4 ± 2.2	0.655
Nulliparity	1355 (37.1%)	10 (38.5%)	1.000
Previous cesarean delivery	157/2197 (7.2%)	3/16 (18.8%)	0.104
Sonographic biometry			
Estimated fetal weight (AC, FL), g	3023.7 ± 731.5	2734.5 ± 353.9	0.334
Estimated fetal weight (AC, BPD), g	3209.8 ± 557.60	2685.8 ± 420.8	**<.001**
Head circumference, mm	329.2 ± 15.4	306.1 ± 10.1	**<.001**
Abdominal circumference, mm	336.2 ± 24.6	323.2 ± 21.2	**<.007**
Femur length, mm	72.5 ± 4.4	72.1 ± 3.7	0.594
Bi-parietal diameter, mm	91.3 ± 5.0	85.4 ± 3.8	**<.001**
Chronic hypertension	28/3257 (0.9%)	0/20 (0%)	1.000
Type 1 or type 2 pre-gestational diabetes	74/3261 (2.3%)	1/20 (5.0%)	0.371

Data are presented and mean and standard deviation for continuous variables and number and percent for categorical variables

*HC* Head circumference, *AC* Abdominal circumference, *FL* Femur length, *BPD* Biparietal diameter

Maternal and neonatal outcome parameters are shown in Tables 2 and 3, respectively. Compared to the reference group, the microcephaly group had a higher percentage of males (90.0% vs. 48.2%, *p* < .001), lower birthweight (2957.0 ± 330.4 vs. 3323.2 ± 432.2 g, *p* < .001), and lower birthweight percentile (33.6 ± 21.5 vs. 60.5 ± 26.4, *p* < .001). There were no between-group differences in mode of delivery, OASIS, NICU admission, intraventricular hemorrhage, neonatal respiratory outcome, and composite outcome.

**Table 2:** Maternal and obstetrical outcomes in 3677 fetuses stratified by head circumference

Parameter	Normal HC	Microcephaly	***p***-Value
**N**	**3651**	**26**	
Gestational diabetes mellitus	503/3312 (15.2%)	1/20 (5.0%)	0.345
Preeclampsia, overall	140/3269 (4.3%)	1/21 (4.8%)	0.603
With severe features	15/3255 (0.5%)	0	1.000
Oligohydramnios	117/3258 (3.6%)	3/20 (15.0%)	**0.035**
Polyhydramnios	105/3263 (3.2%)	0/20 (0%)	1.000
Induction of labor	1552/3651 (42.5%)	14/26 (53.9%)	0.703
Mode of delivery			0.372
Spontaneous vaginal	2993 (82.0%)	22 (81.5%)	
Operative vaginal	426 (11.7%)	4 (14.8%)	
Cesarean, intrapartum	232 (6.3%)	0 (0%)	
Indication			
Non-reassuring fetal heart rate	1214 (53.5%)		
Non progress of labor	101 (43.5%)		
Other	7 (3%)		
Gestational age at delivery, weeks	39.0 ± 1.2	39.3 ± 1.3	0.205
Episiotomy	878/3360 (26.1%)	6/22 (27.3%)	1.000
OASIS	22/3257 (0.7%)	0/20 (0%)	1.000
Shoulder dystocia	15/3256 (0.5%)	1/20 (5.0%)	0.094

Data are presented and mean and standard deviation for continuous variables and number and percent for categorical variables

*OASIS* Obstetrical anal sphincter injury syndrome

**Table 3:** Neonatal outcomes in 3677 fetuses stratified by head circumference

Parameter	Normal HC	Microcephaly	***p***-Value
**N**	**3651**	**26**	
Neonatal sex, male	1230/2552 (48.2%)	18/20 (90.0%)	**<.001**
Neonatal birthweight, g	3323.2 ± 432.2	2957.0 ± 330.4	**<.001**
Neonatal birthweight, centile	60.5 ± 26.4	33.6 ± 21.5	**<.001**
5-min Apgar score < 7	32	0	0.631
Jaundice	420/3651 (11.5%)	5/26 (19.2%)	0.215
Phototherapy	75/3651 (2.1%)	1/26 (3.9%)	0.420
Asphyxia	42/3651 (1.2%)	0	1.000
NICU admission	189/3639 (5.2%)	3/26 (11.5%)	0.153
Seizure	7/3651 (0.2%)	0	1.000
Intraventricular hemorrhage	7/3651 (0/2%)	0	1.000
Transient tachypnea of the newborn	34/3651 (0.9%)	0	1.000
Respiratory distress syndrome	8/3651 (0.2%)	0	1.000
Neonatal sepsis	9/3651 (0.3%)	0	1.000
Composite outcome^a^	241/3651 (6.6%)	3/26 (11.5%)	0.420

Data are presented and mean and standard deviation for continuous variables and number and percent for categorical variables

*NICU* Neonatal intensive care unit

^a^Composite outcome – any one of the following: NICU admission, intraventricular hemorrhage, 5-min Apgar score < 7, asphyxia, seizures, and sepsis

## Discussion

The present study shows that isolated microcephaly in term fetuses has no significant effect on mode of delivery or on maternal and neonatal outcomes.

The rate of microcephaly in our cohort of 3677 women was 0.7% when microcephaly was defined as 2SD below the mean for age and sex. Daniel-Spiegel et al. [17] defined fetal microcephaly as head circumference 3SD below the norm and reported a rate of 0.2% in term pregnancies; however only 0.11% of the neonates included in their study were found to be microcephalic at post-partum evaluation. Subsequently, several studies sought to improve the accuracy of the prenatal detection of microcephaly and reduce the high false-positive rate found after labor. Leibovitz et al. [18] constructed a reference range for a novel biometric measure, the foramen-to-cranium distance (FCD), defined as the distance between the foramen magnum and upper inner cranial border along the posterior wall of the brainstem. When it was applied to 25 fetuses diagnosed with microcephaly, the rate of false positives was reduced. In another study the same group found that the use of two new reference ranges for fetal head circumference [19, 20] did not significantly improve the prediction of postpartum microcephaly over the currently accepted nomogram [4].

Successful vaginal delivery depends on appropriate entrance of the fetal head into the pelvic cavity, subsequent rotational movement of the head to enable its passage through the interspinous distance (the narrowest part of the mid-pelvis), and proper exit of the head from the pelvic outlet, extending through and below the pubic rami. Given that a large head diameter and circumference are known to be associated with higher-than-normal rates of operative vaginal delivery and cesarean delivery [13–16], it might be assumed that a small head diameter would facilitate passage of the fetus through the birth canal, resulting in significantly reduced rates of delivery complications. However, in the present study, the distribution of spontaneous vaginal and vaginal operative deliveries was similar for the microcephalic fetuses and the fetuses with a normal head circumference. This finding suggests that additional factors in labor process may decrease the potential gain of small head circumference, such as a narrow pubic arch angle and small maternal stature, which correlate with a small pelvic diameter [21, 22]. Both these factors have been associated with a higher rate of instrumental vaginal delivery. Be that as it may, our results indicate that a small head as an isolated finding is not a contributory factor in vaginal birth. There were also no intrapartum cesarean deliveries in the study group compared to a 6.3% rate in the reference group. Further studies with a larger sample of microcephalic fetuses are needed to determine if this difference from fetuses with a normal head circumference, although not statistically significant, is attributable to the small number of fetuses with microcephaly in our cohort or in fact to their lower risk of protracted labor (43.5% in the reference group) or non-reassuring fetal heart rate (53.5% in the reference group).

Birth weight was significantly lower in the microcephalic group than the reference group (2957 ± 330 vs. 3323.2 ± 432.1 g), as was the birth weight percentile (33.6 ± 21.5 vs. 60.5 ± 26.4). It is noteworthy that in some cases, a prenatal finding of microcephaly may indicate an overall small fetus and not true microcephaly. Although we excluded small-for-gestational-age newborns, and in utero abdominal circumference was similar in the groups, we defined microcephaly as 2SD below the mean for gestational age. Had we used a cutoff of 3SD, we would have lowered any possible chances of including misdiagnosed small fetuses with no microcephaly-associated pathology**.**

There were no between-group differences in any of the outcome parameters, suggesting that isolated term microcephaly does not pose a risk for adverse perinatal outcome. Thus, considering that only 1 of the 26 fetuses in the microcephaly group had a head circumference below 3SD of the norm, we may assume that microcephaly up to 2SD of the norm is of limited clinical significance.

Our study has several limitations. First, the cohort included only 26 fetuses with isolated microcephaly, although the rate of fetal microcephaly at our center was representative of the general population (0.7%). This may be a cause of type II error - however, it is impractical to reach an appropriate sample size in a single center setting. Second, owing to the retrospective design, we were lacking some potentially relevant information which could have contributed to the analysis of our findings, such as parental head diameter/height and pubic arch angle, in addition to sonographic parameters such as angle of progression and head perineal distance that could have added information about labor progress [23–26]. Also, routine ante-partum fetal weight estimation and biometry is not universally recommended, nor routinely preformed - this may be a source of bias in our study, as well as the fact that truly isolated microcephaly can only be ascertained after birth. Nonetheless, microcephaly is not a common finding, and our selection process helped us to remain focused on the study question while avoiding confounders such as structural anomalies, genetic abnormalities, and growth restriction with a possible influence on perinatal outcome. To the best of our knowledge, this is the first study addressing the impact of a small fetal head on delivery and labor outcome. The findings have potential implications for counseling women prior to delivery of a microcephalic fetus.

## Conclusion

Fetuses with isolated microcephaly have a similar mode of delivery and perinatal outcome to fetuses with a normal head circumference. Further prospective studies that take additional valuable pre-labor and sonographic information into account are needed to better characterize the effect of microcephaly on labor progress and perinatal factors.

## Data Availability

The data that support the findings of this study are available on request from the corresponding author. The data are not publicly available due to privacy or ethical restrictions.

## Abbreviations

BPD: Bi-parietal diameter
CD: Cesarean delivery
HC: Head circumference
NICU: Neonatal intensive care unit
OASIS: Obstetric anal sphincter injury syndrome
OVD: Operative vaginal delivery
SD: Standard deviations

## References

[1] Ashwal S, Michelson D, Plawner L, Dobyns WB (2009). Quality Standards Subcommittee of the American Academy of Neurology and the Practice Committee of the Child Neurology Society. Practice parameter: evaluation of the child with microcephaly (an evidence-based review): report of the quality standards Subcommittee of the American Academy of neurology and the practice Committee of the Child Neurology Society. Neurology.

[2] Woods CG (2004). Human microcephaly. Curr Opin Neurobiol.

[3] Woods CG, Parker A (2013). Investigating microcephaly. Arch Dis Child.

[4] Chervenak FA, Jeanty P, Cantraine F, Chitkara U, Venus I, Berkowitz RL (1984). The diagnosis of fetal microcephaly. Am J Obstet Gynecol.

[5] Basel-Vanagaite L, Dobyns WB (2010). Clinical and brain imaging heterogeneity of severe microcephaly. Pediatr Neurol.

[6] Dolk H (1991). The predictive value of microcephaly during the first year of life for mental retardation at seven years. Dev Med Child Neurol.

[7] Stoler-Poria S, Lev D, Schweiger A, Lerman-Sagie T, Malinger G (2010). Developmental outcome of isolated fetal microcephaly. Ultrasound Obstet Gynecol.

[8] Leibovitz Z, Lerman-Sagie T (2018). Diagnostic approach to fetal microcephaly. Eur J Paediatr Neurol.

[9] Bardin R, Aviram A, Meizner I, Ashwal E, Hiersch L, Yogev Y (2016). Association of fetal biparietal diameter with mode of delivery and perinatal outcome. Ultrasound Obstet Gynecol.

[10] Pavličev M, Romero R, Mitteroecker P (2020). Evolution of the human pelvis and obstructed labor: new explanations of an old obstetrical dilemma. Am J Obstet Gynecol.

[11] Aviram A, Yogev Y, Bardin R, Hiersch L, Wiznitzer A, Hadar E (2016). Association between sonographic measurement of fetal head circumference and labor outcome. Int J Gynaecol Obstet.

[12] Lipschuetz M, Cohen SM, Israel A, Baron J, Porat S, Valsky DV (2018). Sonographic large fetal head circumference and risk of cesarean delivery. Am J Obstet Gynecol.

[13] Kabiri D, Lipschuetz M, Cohen SM, Yage O, Levitt L, Herzberg S (2019). Vacuum extraction failure is associated with a large head circumference. J Matern Fetal Neonatal Med.

[14] Seltzer LE, Paciorkowski AR (2014). Genetic disorders associated with postnatal microcephaly. Am J Med Genet C Semin Med Genet.

[15] Barkovich AJ, Guerrini R, Kuzniecky RI, Jackson GD, Dobyns WB (2012). A developmental and genetic classification for malformations of cortical development: update 2012. Brain..

[16] Dollberg S, Haklai Z, Mimouni FB, Gorfein I, Gordon E-S (2005). Birth weight standards in the live-born population in Israel. Isr Med Assoc J.

[17] Daniel-Spiegel E, Weiner E, Yarom I, Doveh E, Friedman P, Cohen A (2013). Establishment of fetal biometric charts using quantile regression analysis. J Ultrasound Med.

[18] Leibovitz Z, Shiran C, Haratz K, Tamarkin M, Gindes L, Schreiber L (2016). Application of a novel prenatal vertical cranial biometric measurement can improve accuracy of microcephaly diagnosis in utero. Ultrasound Obstet Gynecol.

[19] Leibovitz Z, Daniel-Spiegel E, Malinger G, Haratz K, Tamarkin M, Gindes L (2016). Prediction of microcephaly at birth using three reference ranges for fetal head circumference: can we improve prenatal diagnosis?. Ultrasound Obstet Gynecol.

[20] Apageorghiou AT, Ohuma EO, Altman DG, Todros T, Cheikh Ismail L, Lambert A (2014). International standards for fetal growth based on serial ultrasound measurements: the Fetal Growth Longitudinal Study of the INTERGROWTH-21st Project. Lancet..

[21] Gilboa Y, Kivilevitch Z, Spira M, Kedem A, Katorza E, Moran O (2013). Pubic arch angle in prolonged second stage of labor: clinical significance. Ultrasound Obstet Gynecol.

[22] Youssef A, Ghi T, Martelli F, Montaguti E, Salsi G, Bellussi F (2016). Subpubic arch angle and mode of delivery in low-risk nulliparous women. Fetal Diagn Ther.

[23] Henrich W, Dudenhausen J, Fuchs I, Kämena A, Tutschek B (2006). Intrapartum translabial ultrasound (ITU): sonographic landmarks and correlation with successful vacuum extraction. Ultrasound Obstet Gynecol.

[24] Tutschek B, Braun T, Chantraine F, Henrich W (2011). A study of progress of labour using intrapartum translabial ultrasound, assessing head station, direction, and angle of descent. BJOG..

[25] Tutschek B, Torkildsen EA, Eggebø TM (2013). Comparison between ultrasound parameters and clinical examination to assess fetal head station in labor. Ultrasound Obstet Gynecol.

[26] Ghi T, Farina A, Pedrazzi A, Rizzo N, Pelusi G, Pilu G (2009). Diagnosis of station and rotation of the fetal head in the second stage of labor with intrapartum translabial ultrasound. Ultrasound Obstet Gynecol.

